# The Nuclear Receptor DAF-12 Regulates Nutrient Metabolism and Reproductive Growth in Nematodes

**DOI:** 10.1371/journal.pgen.1005027

**Published:** 2015-03-16

**Authors:** Zhu Wang, Jonathan Stoltzfus, Young-jai You, Najju Ranjit, Hao Tang, Yang Xie, James B. Lok, David J. Mangelsdorf, Steven A. Kliewer

**Affiliations:** 1 Deparment of Pharmacology, University of Texas Southwestern Medical Center at Dallas, Dallas, Texas, United States of America; 2 Department of Pathology, School of Veterinary Medicine, University of Pennsylvania, Philadelphia, Pennsylvania, United States of America; 3 Department of Molecular Biology, University of Texas Southwestern Medical Center at Dallas, Dallas, Texas, United States of America; 4 Department of Clinical Science, University of Texas Southwestern Medical Center at Dallas, Dallas, Texas, United States of America; 5 Howard Hughes Medical Institute, University of Texas Southwestern Medical Center at Dallas, Dallas, Texas, United States of America; Harvard University, UNITED STATES

## Abstract

Appropriate nutrient response is essential for growth and reproduction. Under favorable nutrient conditions, the *C*. *elegans* nuclear receptor DAF-12 is activated by dafachronic acids, hormones that commit larvae to reproductive growth. Here, we report that in addition to its well-studied role in controlling developmental gene expression, the DAF-12 endocrine system governs expression of a gene network that stimulates the aerobic catabolism of fatty acids. Thus, activation of the DAF-12 transcriptome coordinately mobilizes energy stores to permit reproductive growth. DAF-12 regulation of this metabolic gene network is conserved in the human parasite, *Strongyloides stercoralis*, and inhibition of specific steps in this network blocks reproductive growth in both of the nematodes. Our study provides a molecular understanding for metabolic adaptation of nematodes to their environment, and suggests a new therapeutic strategy for treating parasitic diseases.

## Author Summary

Animals adjust their internal biological processes in response to their environments. In this study, we report that in a nutrient rich environment the free-living nematode, *Caenorhabditis elegans*, induces an energy-generating metabolic pathway to govern its reproductive growth by activating the nuclear receptor, DAF-12. By responding to its endogenous ligands, called dafachronic acids, DAF-12 induces oxidation of lipids to produce the energy necessary to support growth and reproduction; and likewise, in the absence of dafachronic acids, DAF-12 prevents activation of this pathway. Through gene expression analysis, we show that DAF-12 regulates a network of genes involved in energy homeostasis and lipid metabolism. Given that dafachronic acids are produced only in well-fed worms, we conclude that DAF-12 functions as an environmental sensor that coordinately governs energy homeostasis. Through analogous studies in the incurable human parasite, *Strongyloides stercoralis*, we demonstrate that this pathway is conserved and that blocking it compromises the viability of the parasites. These findings elucidate a molecular mechanism for how nematodes govern their energy needs in response to the environment, and provide a potential new strategy for treating nematode parasitic diseases.

## Introduction

The evolutionary success of nematodes is derived from their ability to adapt different developmental pathways depending on environmental conditions. The best studied of these pathways exists in the free-living nematode *Caenorhabditis elegans (C*. *elegans)*. After hatching from eggs when enviromental conditions are favorable, *C*. *elegans* larvae continually develop through four stages (L1–L4), which eventually mature into reproductive adults. In contrast, in unfavorable environments, *C*. *elegans* larvae interrupt their reproductive growth by arresting at an alternative L3 stage termed dauer, which is characterized by developmental quiescence, stress resistance and a substantial extension of lifespan. Once conditions become favorable, the L3-dauers exit the developmental diapause and rapidly progress into the L4 stage through a series of metabolic and developmental changes that are governed by a coordinated transcriptional network [1]. Through this developmental adjustment, *C*. *elegans* is able to maximize its reproductive advantage under diverse environmental conditions [2]. Similar to free-living species like *C*. *elegans*, parasitic nematodes also alter their larval development based on environmental conditions [3,4]. Before host infection occurs, larvae of developing parasitic nematodes, such as hookworms and *Strongyloides stercoralis (S*. *stercoralis)*, arrest their reproductive growth at a dauer-like stage called infectious L3 (iL3). Then, upon infection of their hosts where environmental conditions favor the completion of the parasite’s life cycle, the arrested iL3 larvae resume reproductive growth and develop into fertile egg-laying adults.

At the molecular level, the nematode development program is controlled by a hormonal signaling pathway initiated by insulin/IGF-I and TGF-β, which eventually converges in the activation of a nuclear receptor called DAF-12 [2,5,6]. In *C*. *elegans*, favorable environments stimulate insulin/IGF-I and TGF-β pathways that induce expression of DAF-9, a cytochrome P450 enzyme that catalyzes the synthesis of steroid-like hormones, called dafachronic acids [2,5,6]. Dafachronic acids serve as ligands that bind and activate DAF-12 [5–7], which in turn commit the nematode to reproductive growth. Conversely, in unfavorable environments, the insulin/IGF-I and TGF-β pathways remain inactive, preventing dafachronic acid synthesis, which in turn allows DAF-12 to interact with DIN-1, a strong co-repressor that is required for dauer formation [5,6,8]. In *C*. *elegans* larvae lacking DAF-12, this repressor activity is absent, causing a dauer-defective phenotype that would be expected to decrease viability in an unfavorable environment [2,9]. In parasitic nematodes, the insulin/IGF-I/DAF-12 signaling pathway controlling development appears to be conserved [10–15]. Similar to *C*. *elegans*, in parasitic nematodes ligand-free DAF-12 is required for formation of the dauer-like iL3, whereas ligand-activated DAF-12 is required for reproductive growth [15].

In *C*. *elegans*, larvae undergoing reproductive growth or dauer diapause display distinct patterns of energy metabolism. L2–L4 larvae in reproductive growth exhibit aerobic energy metabolism by converting dietary energy sources (carbohydrates and lipids) into acetyl-CoA, which is then fed into the TCA cycle and oxidative phosphorylation [2,16,17]. This aerobic metabolism produces sufficient energy to support rapid, energy-demanding reproductive growth. In contrast, aerobic energy metabolism is greatly reduced in dauer larvae, which instead exhibit a slower rate of anaerobic energy metabolism. Paradoxically, however, anaerobic metabolism also utilizes fat metabolism to meet the nematode’s energy needs for survival during privation [18–21]. The pathways involved in anaerobic energy metabolism are the glyoxylate cycle, a variant of the TCA cycle that converts acetyl-CoA to malate, and malate dismutation, a fermentation pathway that metabolizes malate for energy production [2,16,17]. The tendency towards the lower rate of anaerobic metabolism in dauer larvae prevents premature depletion of energy reserves and facilitates extended lifespan [22]. Thus, two distinct types of metabolism are employed to produce energy during reproductive growth and diapause stages, raising the question as to how the two pathways are differentially regulated.

Although the study of metabolism in parasitic nematodes is hampered by the difficulty in obtaining sufficient numbers, it is known that during reproductive growth in their hosts certain species of parasitic larvae migrate through the circulatory system, lungs and trachea, where aerobic conditions are high [3,4]. Furthermore, iL3 larvae of the hookworm *Ancylostoma caninum* are suggested to use fat reserves as an energy source [23], and there is an inverse correlation between oxygen consumption and iL3 longevity in parasitic nematodes [24]. These findings suggest that similar mechanisms control developmental energy metabolism in free-living and parasitic nematodes.

In the present study, we show that in addition to governing expression of developmental genes required for entry and exit from dauer diapause, DAF-12 is required for activating a metabolic network that is required for the normal progression to reproductive maturity. Efforts to elucidate the molecular targets of DAF-12 have focused mainly on the identification of heterochronic and microRNA genes that ensure the correct developmental decision is made during entrance and exit from dauer [25–28], and on longevity genes that are repressed in long-lived mutant worms [29–31]. Notably, however, a role for DAF-12 in energy homeostasis has not been well documented. Utilizing a combination of biochemical and genetic approaches, we show that DAF-12 is a key transcriptional regulator of developmental energy metabolism. In *C*. *elegans*, DAF-12 induces expression of a gene network that is responsible for aerobic fat utilization during reproductive growth. Further, this DAF-12-dependent metabolic network is conserved in the parasitic nematode, *S*. *stercoralis*. This work provides a molecular understanding of how nematodes adjust energy metabolism to assure successful reproduction in wide-ranging environments, and it suggests a therapeutic strategy for treating parasitic diseases by inhibiting fat utilization.

## Results

### DAF-12 stimulates aerobic fatty acid metabolism and reproductive growth

**Fig 1:**
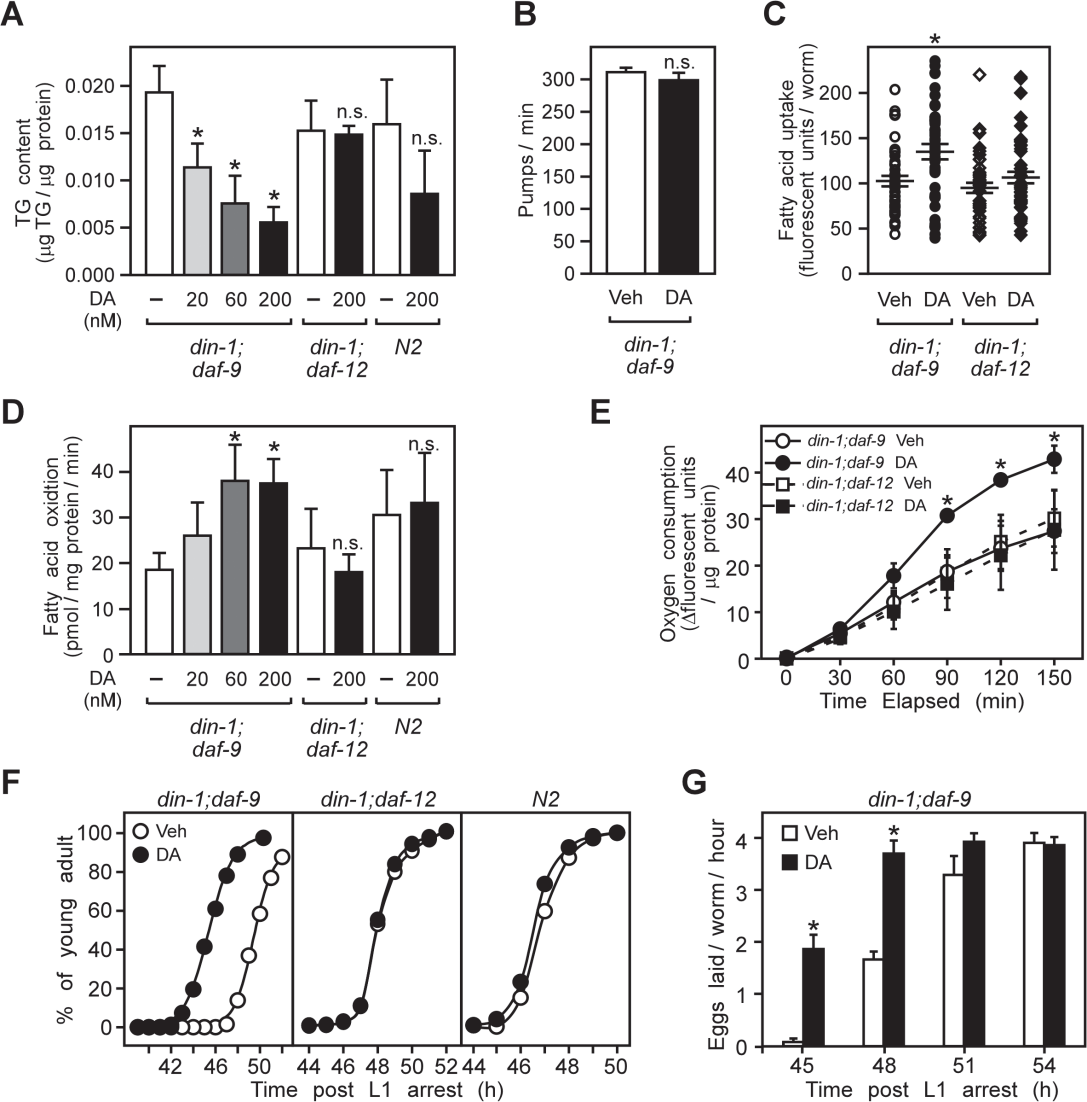
DAF-12 activation promotes aerobic lipid metabolism and reproductive growth in *C*. *elegans*. (A) Triglyceride (TG) content, (B) pharyngeal pumping rates, (C) dietary fatty acid uptake, (D) fatty acid oxidation and (E) oxygen consumption in L3 larvae that were synchronized and treated from the L1 stage for 22.5 h at 25°C with vehicle or DA at the indicated concentration (A, D), or 200 nM (B, C, E). (F,) Stimulation of reproductive growth as evaluated by the percentage of L4 larvae that become adults after treatment of synchronized L1 larvae with vehicle or 200 nM DA. (G) Time to egg-laying after treatment of synchronized L1 larvae with vehicle or 200 nM DA. N = 3 ± S.D.; * *P* < 0.05 comparing vehicle to DA treatment by Student’s t-test in (A–E, G); n.s., not significant. In (C), error bars = S.E.M. Vehicle, ethanol; DA, Δ7-dafachronic acid.

To investigate the potential role of DAF-12 in regulating energy metabolism in *C*. *elegans*, we used a dauer defective double-null mutant lacking both the DAF-12 co-repressor *din-1* and the dafachronic acid-synthesizing enzyme *daf-9* [8]. Employing a mutant that lacks both *din-1* and *daf-9* commits *C*. *elegans* to constitutive reproductive growth even in the absence of dafachronic acids. The advantage of the *din-1;daf-9* mutant is that it permits evaluation of the direct effects of DAF-12 activation on metabolism while at the same time minimizing effects due to the developmental switching that would otherwise occur in the single null mutant of *daf-9*. We found that treating *din-1;daf-9* larvae with the high affinity endogenous ligand, Δ7-dafachronic acid (DA) decreased triglyceride levels in a dose dependent manner (Fig. 1A). This decrease was not due to reduced dietary nutrient uptake, since DA treatment had no effect on pharyngeal pumping rates of the larvae (Fig. 1B) but rather slightly increased dietary fatty acid uptake (Fig. 1C). In contrast, DA treatment increased the fatty acid oxidation and oxygen consumption in *din-1;daf-9* larvae (Fig. 1D, E). At the same time, DA treatment did not significantly change either triglyceride levels, fatty acid oxidation, or oxygen consumption in mutants that lack DAF-12 (*din-1;daf-12*) or in wild type *N2* larvae (Fig. 1A, D, E), indicating that the effects of DA on metabolism were DAF-12 dependent.

We also examined whether DAF-12 activation affects reproduction. As shown in Fig. 1F, progression from L4 to the young adult stage, when reproductive organs become well-developed [32], occurred earlier in *din-1;daf-9* larvae treated with DA compared to vehicle in a DAF-12 specific manner. DA treatment also advanced the onset of egg laying, another marker of reproductive maturity (Fig. 1G). Together, these findings demonstrate that DAF-12 activation induces aerobic energy metabolism and accelerates larval reproductive growth.

### DAF-12 regulates genes involved in fatty acid metabolism

To gain insight into the molecular mechanism underling the DAF-12-regulated fatty acid metabolism, we evaluated global changes in *C*. *elegans* gene expression by comparing vehicle and DA treated L3 larvae. Microarray analysis identified 796 genes that were up-regulated and 985 genes that were down-regulated (>2-fold change and FDR<5%) in response to DAF-12 activation (S1 Table). The DA-regulated transcriptome was then grouped into several functional categories based on gene ontology (DAVID, ref. [33], S1 Table). In addition to the expected changes in expression of heterochronic and molting genes (e.g., *dre-1*) that coordinately regulate developmental and reproductive pathways [26], DA governed expression of a distinct cadre of genes involved in the metabolism of lipids (S1 Table). In contrast, no changes were observed in the expression of genes required for metabolizing glucose.

We then compared the microarray data from our DA responsive genes with that of genes that have been shown to be regulated during the exit from dauer [1], which is another process where DAF-12 is activated. As expected, the transcriptome of DA up-regulated genes significantly overlapped the trancriptome of genes up-regulated during dauer recovery (S1A Fig.). These data indicate that DAF-12 engages a gene network that governs metabolism and growth during both reproductive development and dauer recovery. We also compared the DAF-12 transcriptome with genes that are regulated by the transcription factor DAF-16 [34]. Whereas DAF-12 activation suppresses dauer, activation of DAF-16 is known to promote dauer [2]. As expected by this reciprocal regulation of dauer, there was no statistically significant overlap between genes regulated by DAF-12 and DAF-16 (S1B Fig. and S1C Fig.). Although this comparison was between the transcriptomes from different stages of worms (L3 for DAF-12 vs. adult for DAF-16), these results suggest that DAF-12 and DAF-16 regulate distinct gene networks to coordinate entry and exit from dauer diapause, and the initiation of metabolic pathways that promote reproductive development.

**Fig 2:**
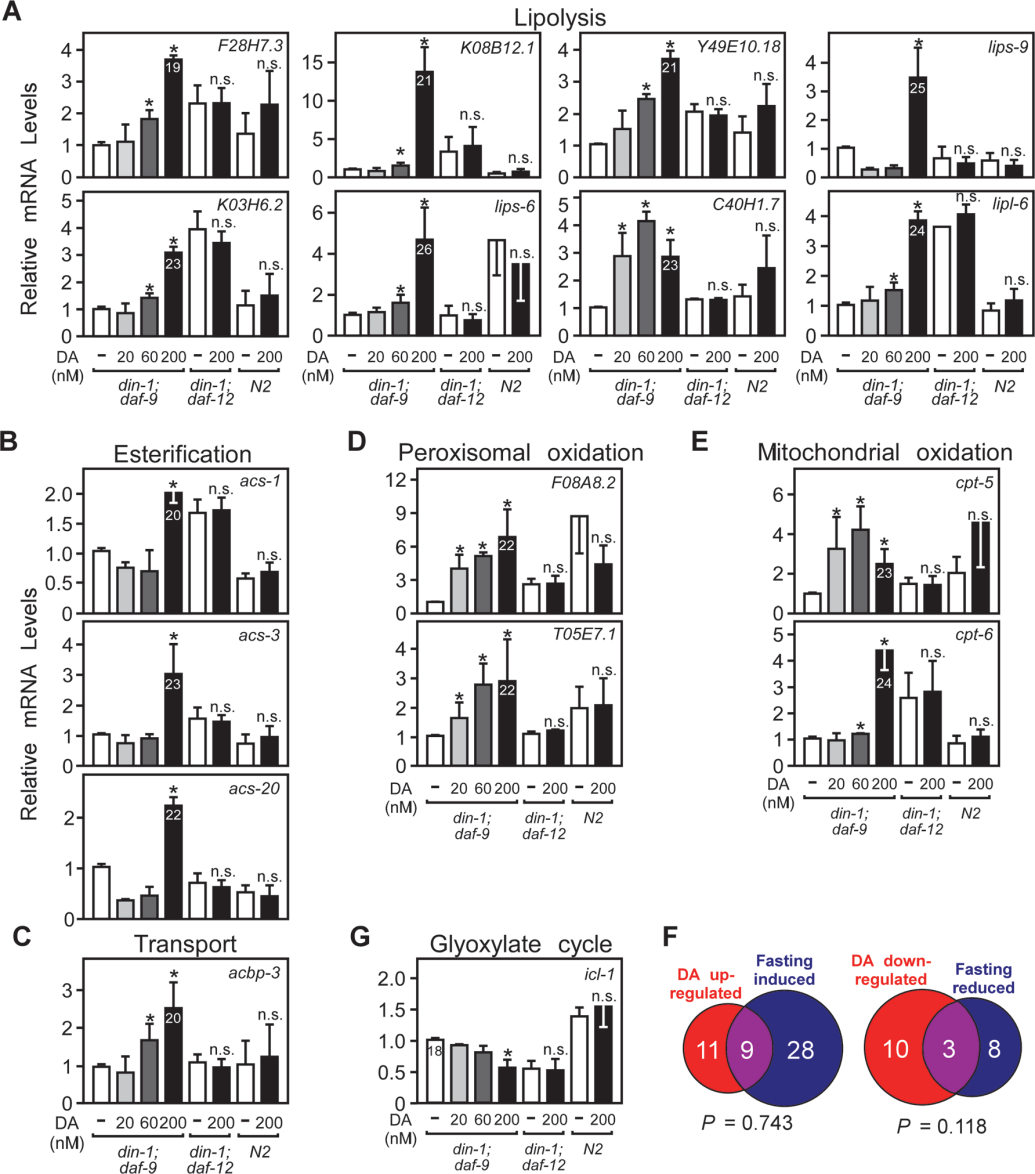
Regulation of fat utilization genes by DAF-12 in *C*. *elegans*. (A–E, G) Fatty acid metabolic gene expression in L3 worms. Synchronized L1 larvae were grown in the presence of vehicle or the indicated concentrations of DA for 22.5 hrs and mRNA expression was quantitated by qPCR. Numbers in bars refer to Ct values. (F) Comparison of DAF-12 and fasting regulated metabolic genes. *P< 0.05 by Student’s t-test comparing vehicle to DA treatment *(*n = 4 ± S.D.), n.s., not significant. Vehicle, ethanol; DA, Δ7-dafachronic acid.

To further investigate the metabolic network governed by DAF-12, we used qPCR to confirm changes in expression of fatty acid metabolic genes identified in our microarray study, as well as other candidate genes known to be involved in fatty acid metabolism [19,20]. A total of 69 genes were evaluated (S2 Table). A hallmark of the DA-regulated metabolic pathway that correlates to reproductive growth is the induction of aerobic fatty acid oxidation (Fig. 1). DA increased expression of genes involved in every aspect of aerobic fatty acid utilization, including lipolysis, transport, esterification, and oxidation in both peroxisomes and mitochondria (Fig. 2A-E; S2 Table).

To provide an additional objective assessment of DAF-12’s role in regulating energy metabolism, we compared the DA-regulated lipid metabolic gene profile to changes observed in response to fasting. In addition to reproductive growth, fasting is another physiological process that mobilizes and utilizes fatty acids [19,20]. However, in contrast to reproductive growth, fasting utilizes anaerobic metabolism marked by reduced metabolic rates [21] and activation of the glyoxylate cycle (through *icl-1* expression) [19,20]. Of the 69 fatty acid metabolic genes tested above (S2 Table), 20 were increased by DAF-12 and 37 were increased by fasting (Fig. 2F, S2 Table). Importantly, there was no significant overlap (based on hypergeometric distribution) in the number of genes that were either up- or down-regulated under both conditions, demonstrating that DAF-12 and fasting engage distinct gene networks for fatty acid utilization. DA decreased the mRNA levels of *icl-1* (Fig. 2G), the bi-functional enzyme with isocitrate lyase and malate synthase activities that is unique to the glyoxlate cycle and thus serves for an indicator of anaerobic fatty acid utilization. Taken together with the biochemical measurements of fatty acid oxidation (Fig. 1), these results suggest that DAF-12 activation selectively governs the pathways that lead to energy mobilization and utilization by increasing expression of genes involved in aerobic lipid metabolism.

We also investigated the signaling pathways that govern metabolism of DA. Interestingly, expression of *daf-28* (insulin/IGF-I-like), *daf-7* (TGF-β-like) and *daf-9* (the DA biosynthesis enzyme) were specifically suppressed by exogenous DA treatment, while expression of *strm-1*, which quenches DAF-12 ligand synthesis [35], was induced (S2 Fig.). The ability of DAF-12 to repress its own activity is reminiscent of a classic endocrine feedback circuit that functions to maintain homeostasis.

**Fig 3:**
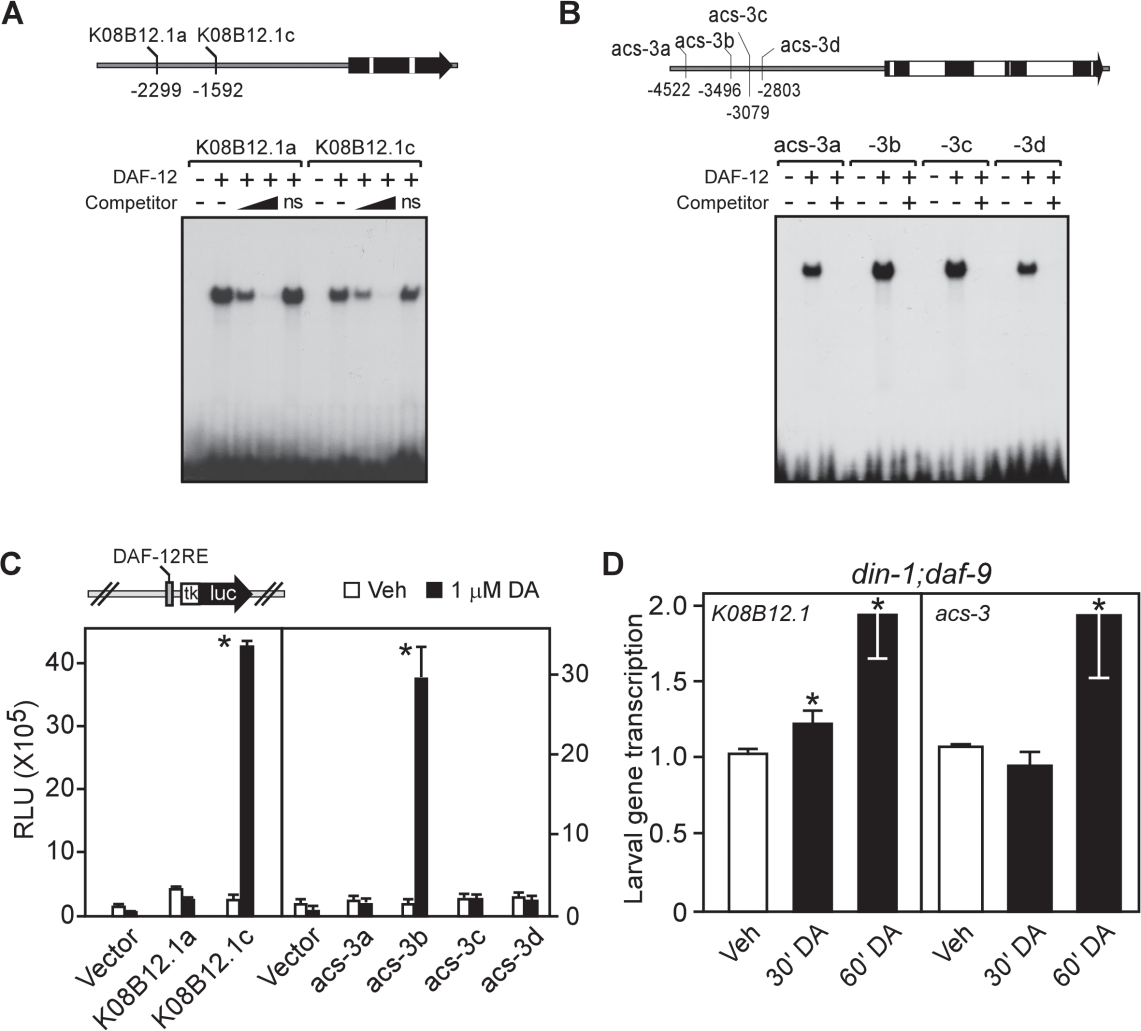
Fat utilization genes are direct targets of *C*. *elegans* DAF-12. (A, B) Gel electrophoresis mobility shift assays with [^32^P]-labelled oligonucleotides corresponding to each of the candidate DAF-12 response elements (shown in the schematics of the promoters above the panels) for TG lipase (*K08B12*.*1*) and acyl-CoA synthase (*acs-3*). Gel shifts were performed in presence or absence of in vitro translated DAF-12 protein ± the presence of 20-fold and 200-fold (in A) or 200-fold (in B) excess unlabeled competitor oligonucleotides. ns, non-specific oligo at 200-fold excess. (C) Luciferase reporter assays with candidate response elements shown in (A,B) from HEK293 cells cotransfected with reporter and DAF-12 expression plasmids and treated with vehicle or 1 μM DA (n = 3 ± S.D.). (D) Nuclear run-on assays of *K08B12*.*1* and *acs-3* transcription in L2 larvae of *din-1;daf-9(dh127;dh6)* worms transiently treated by vehicle or 500 nM DA (n = 3 ± S.D.). *P< 0.05 comparing vehicle to DA treatment by Student’s t-test. Vehicle, ethanol; DA, Δ7-dafachronic acid.

To determine whether the up-regulation of gene transcription by DA was through the direct action of DAF-12, we analyzed the promoters of several of the DA-induced genes that were confirmed by qPCR. In the absence of a DAF-12 antibody to perform chromatin immunoprecipitation experiments, we used bioinformatics to search for the consensus DAF-12 DNA binding element [36] in the promoters/introns of five representative DAF-12-induced genes. Selection of these genes was based on their representation of different metabolic processes, their high levels of expression, their response to DA, and their distinct chromosomal locations (i.e., genes not likely to be in a gene cluster sharing a common promoter). Our analysis revealed 33 putative DAF-12 response elements (S3 Table). We found that DAF-12 bound efficiently to 13 of these elements (Fig. 3A, B; S3 Table) and activated transcription in a standard cell-based reporter assay through four of them (Fig. 3C; S3 Table). These four DAF-12 response elements corresponded to three (*K08B12*.*1*, *acs-1* and *acs-3*) of the five genes originally selected for analysis. Consistent with these genes being direct transcriptional targets of DAF-12, *K08B12*.*1* and *acs-3* expression was induced rapidly within 30 to 60 minutes after treatment of *din-1;daf-9* larvae with DA (Fig. 3D). These data suggest that at least a portion of the genes regulated by DAF-12 are likely to be direct targets.

### Normal reproductive growth requires DAF-12 to regulate fat utilization

**Fig 4:**
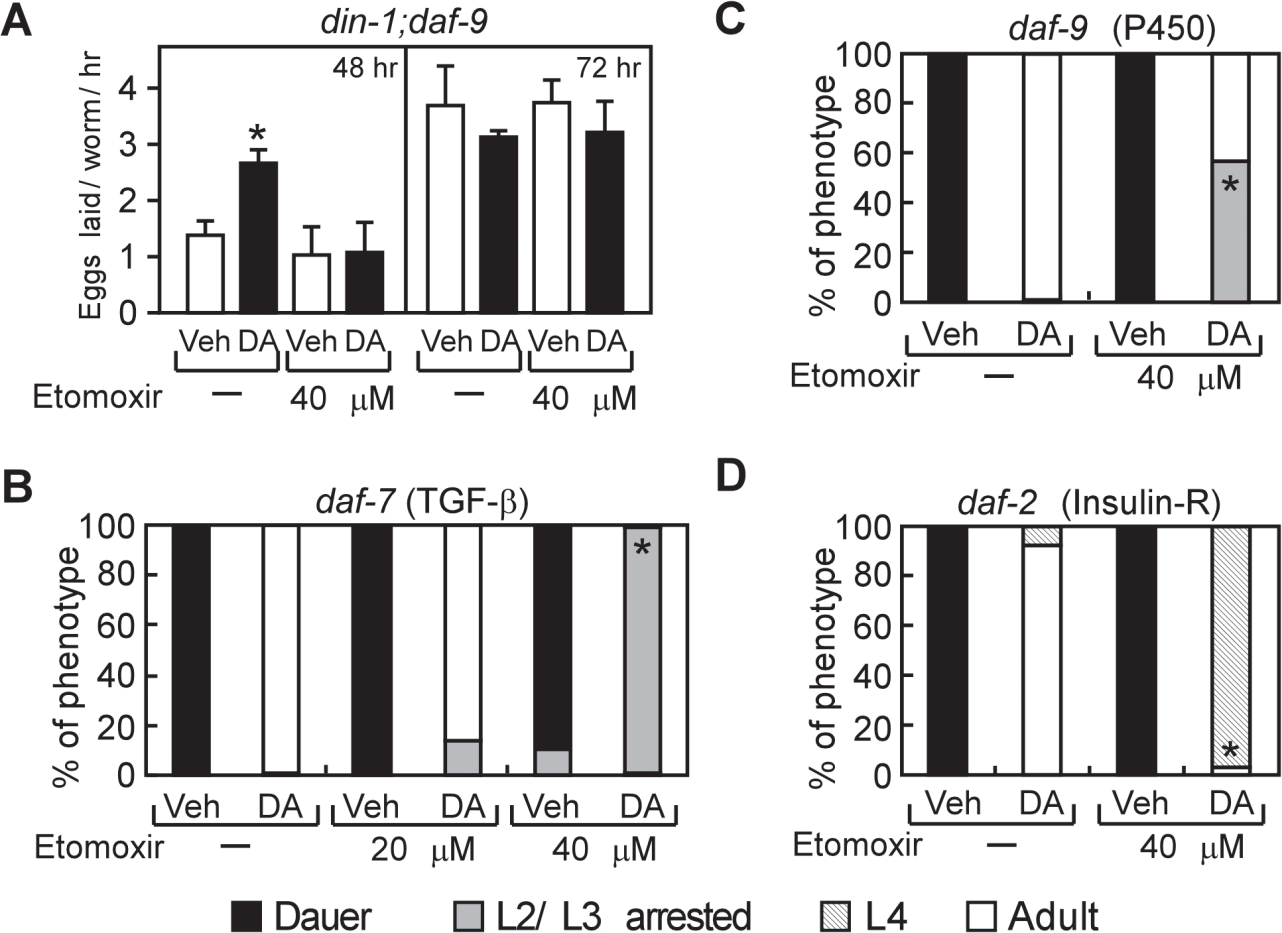
Aerobic lipid metabolism is required for DAF-12 to promote reproductive growth in *C*. *elegans*. (A) Inhibition of fatty acid oxidation by etomoxir delays the onsets of egg laying in *din-1;daf-9* mutants (n = 4), and (B–D) decreases DA-induced reproductive growth in *daf-7(e1372)*, *daf-9(dh6)*, and *daf-2(e1368)* mutants (n = 3). Synchronized L1 larvae were grown with vehicle or 200 nM DA treatment in the presence or absence of etomoxir at 25°C for 48 h or 72 h (in A) or for 60 h (in B–D) and then phenotyped. *P < 0.05 by Student’s t-test comparing vehicle to DA-treated worms (A) or by Fisher’s exact test comparing DA-treated worms ± etomoxir (B–D). Vehicle, ethanol; DA, Δ7-dafachronic acid.

We next asked whether aerobic fatty acid utilization is required for DAF-12 to promote reproductive growth by inhibiting aerobic fatty acid utilization with etomoxir, a specific inhibitor of the carnitine palmitoyltransferases that mediate fatty acid transport into mitochondria [37]. Etomoxir treatment significantly decreased fatty acid oxidation and increased fat storage in *C*. *elegans* (S3 Fig.), demonstrating the effectiveness of the drug in inhibiting this pathway in nematodes. A further consequence of etomoxir treatment was that it completely blocked the earlier onset of egg laying that is dependent on DA, which is a marker for reproductive growth (Fig. 4A). Etomoxir treatment also prevented DA-mediated rescue of reproductive growth in the *daf-7* and *daf-9* mutants (Fig. 4B, C) and delayed the rescued growth in the *daf-2* mutant (Fig. 4D). In this latter mutant, the inability of etomoxir to inhibit growth completely is likely due to compensatory anaerobic fatty acid utilization that is known to occur in the *daf-2* mutants [34,38]. Consistent with the data shown in Fig. 1F, DA treatment did not affect reproductive capacity in wild type *N2* worms or in mutants lacking DAF-12 expression (*din-1;daf-12*, *daf-9;daf-12*, and *daf-7;daf-12*), regardless of the absence or presence of etomoxir (S4 Fig.). In sum, these data demonstrate that aerobic fatty acid metabolism is required for DAF-12 to promote growth from larvae to reproductive adults in *C*. *elegans*.

### DAF-12 regulates fat utilization and reproductive growth in *S*. *stercoralis*

**Fig 5:**
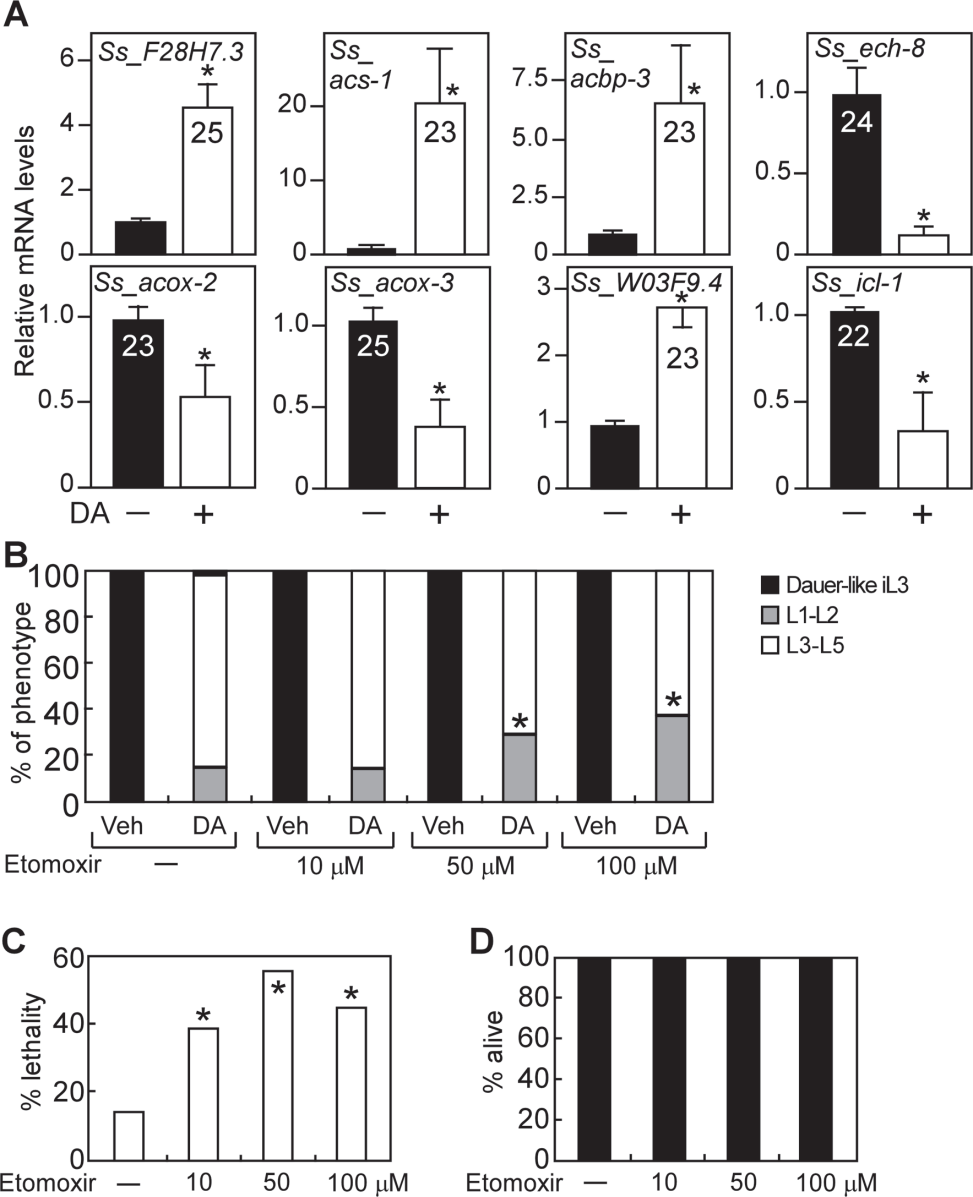
DAF-12-dependent regulation of lipid metabolism is conserved in *S*. *stercoralis*. (A) *F28H7*.*3* (lipase), *acs-1* (synthase), *acbp-3* (transport), *W03F9*.*4* (mitochondrial β-oxidation), *ech-8*, *acox-2* and *acox-3* (perioxisomal β-oxidation), and *icl-1* (glyoxylate cycle) mRNAs were determined by qPCR from *S*. *stercoralis* iL3 larvae treated with or without 400 nM DA. Numbers in bars refer to Ct values. *P-value < 0.05 by Student’s t-test comparing DA to vehicle (n = 3 ± S.D.). (B, C) Effect of the mitochondrial fatty acid oxidation inhibitor, etomoxir, on reproductive development (B) and viability (C) in *S*. *stercoralis* post-free-living larvae treated with DA. *P < 0.05 by Fisher’s exact test comparing larvae treated with 300 nM DA ± etomoxir at the indicated concentrations (n = 3). (D) Etomoxir treatment does not cause lethality in developmentally arrested *S*. *stercoralis* iL3 larvae (n = 3). Vehicle, ethanol; DA, Δ7-dafachronic acid.

Like *C*. *elegans*, many species of parasitic nematodes such as *S*. *stercoralis* also use the conserved insulin/IGF-I and DAF-12 signaling pathways to regulate their development [10–15]. We therefore asked whether the role of DAF-12 in promoting fatty acid utilization is conserved during reproductive growth of *S*. *stercoralis*. To test this, we confirmed the presence of fat utilization genes in *S*. *stercoralis* and then examined whether they are regulated by DA (Fig. 5A, S4 Table). As in *C*. *elegans*, expression of genes encoding a lipase (*Ss_F28H7*.*3*), acyl-CoA synthase (*Ss_acs-1*) and a gene involved in acyl-CoA transport (*Ss_acbp-3*) was induced, while the key glyoxylate cycle gene (*Ss_icl-1*) was repressed by DA treatment. Expression of the carnitine palmitoyltransferase gene, *Ss_W03F9*.*4*, required for mitochondrial β-oxidation, was also increased by DA (Fig. 5A; S4 Table). In contrast, expression of genes involved in peroxisomal β-oxidation (*Ss_acox-2*, *Ss_acox-3* and *Ss_ech-8*) was repressed by DA (Fig. 5A; S4 Table). This profile of gene regulation supports the notion that DAF-12 activation induces aerobic fat utilization in *S*. *stercoralis* similar to that observed for *C*. *elegans* (Fig. 2).

We also examined the effect of etomoxir on DA-induced reproductive growth in the post-free-living larvae of *S*. *stercoralis*, which typically arrest at the dauer-like iL3 stage. Although DA is less potent as an agonist for DAF-12 in *S*. *stercoralis* compared to *C*. *elegans* [15], DA treatment was able to induce >85% maturation of *S*. *stercoralis* larvae to the L3-L5 stages (Fig. 5B). Notably, co-treatment of etomoxir with DA resulted in a significant decrease in the number of L3-L5 larvae (Fig. 5B). Administration of etomoxir to DA-treated worms also led to a marked increase in lethality of these L3-L5 larvae (Fig. 5C). Importantly, treatment with etomoxir alone did not kill *S*. *stercoralis* iL3 larvae (which are in an arrested developmental stage), demonstrating that the lethality observed in L3-L5 worms is due to the specific effects of etomoxir on the reproductive developmental program induced by DA (Fig. 5D).

These results reveal that aerobic fatty acid metabolism is required for DAF-12-dependent reproductive growth of *S*. *stercoralis* and suggest that control of this metabolic pathway by DAF-12 might be a promising strategy for regulating development in these important pathogens.

## Discussion

The nuclear receptor DAF-12 plays an essential role in C. *elegans* in linking nutritional status to developmental programs, including the transition from second- to third-stage larval development and the progression into and out of dauer diapause. In this report, we show that activation of DAF-12 by its hormonal ligand DA directly regulates energy homeostasis by inducing aerobic fatty acid utilization, which is required for reproductive growth. DAF-12 was shown previously to regulate heterochronic genes, which determine the stage-specific timing of cell type differentiation [25–27]. Interestingly, the DAF-12 gene regulatory network is distinct from that of DAF-16, which is active in the absence of DA signaling to promote dauer and inhibit reproductive growth. Thus, DAF-12 controls the balance between reproductive growth and dauer both by controlling the expression of genes directing development, and by regulating the flux of nutrients required to fuel the developmental program. Our findings provide a molecular explanation for the longstanding observation that *C*. *elegans* switches to aerobic metabolism once reproductive growth has been initiated [2,16].

Under favorable environmental conditions, which promote the biosynthesis of DA, we showed that DAF-12 stimulates aerobic fatty acid utilization in larvae as evidenced by decreased storage of triglycerides and increased oxygen consumption and fatty acid oxidation (Fig. 1A, D, E). Although a previous study originally suggested DAF-12 might increase rather than decrease fat storage [8], it has been shown since that the lipid staining assay used in this previous study is non-specific and ineffective for determining fat content [39]. DA treatment also stimulates progression from L4 to the young adult stages and advances the onset of egg-laying activity (Fig. 1F, G). Thus, DAF-12 coordinates the release of energy required to support the rapid, energy-intensive growth of larvae to reproductive maturity. At the molecular level, DA induces metabolic genes that regulate fatty acid utilization at multiple steps, including fatty acid mobilization, esterification, and peroxisomal and mitochondrial β-oxidation. The rate-limiting step enzyme in fatty acid oxidation, carnitine palmitoyltransferase, is encoded by several homologs in *C*. *elegans*, two of which are DAF-12 targets (Fig. 2E) and inhibition of the enzyme’s activity blocks DA-stimulated reproductive growth (Fig. 4). Concomitant with its regulation of genes involved in oxidative metabolism, DA represses the expression of *icl-1*, which encodes a bifunctional enzyme in the glyoxylate cycle that is essential for anaerobic fatty acid catabolism (Fig. 2G). These results support the role of DAF-12 in promoting reproductive growth of *C*. *elegans* by acting as a key controller of energy homeostasis in response to nutrient supply.

The adaptive response to fasting is a process that also mobilizes fat storage to maintain energy homeostasis. However, in contrast to the metabolic pathway involved in reproductive growth, fasting results in an increase in anaerobic metabolism. Fasting decreases metabolic rate [21] and utilizes the glyoxylate cycle to provide energy from fatty acids [18–20], which is diametric to the action of DA. The alternate role of DA in aerobic metabolism is supported by the finding that the metabolic gene networks induced by DAF-12 and by the fasting response are distinct (Fig. 2G). Interestingly, the metabolic response to fasting is mediated by another nematode nuclear receptor, NHR-49 [19,20]. Analogous to the role of DAF-12 in the dauer diapause, NHR-49 is required for the entry and exit of adult reproductive diapause, a process that preserves reproduction in *C*. *elegans* during starvation [40]. Thus, DAF-12 and NHR-49 appear to control two separate gene networks that alternatively use aerobic and anaerobic fatty acid utilization to ensure successful reproduction under varying environmental conditions.

Another key finding of the current study is that DAF-12-mediated regulation of energy homeostasis is conserved in the human parasite, *S*. *stercoralis*. As in *C*. *elegans*, DAF-12 activation stimulates the expression of genes involved in aerobic fatty acid utilization (Fig. 5A). While genes involved in peroxisomal β-oxidation were induced in *C*. *elegans* and repressed in *S*. *stercoralis*, we note that peroxisomal β-oxidation can support either aerobic or anaerobic fatty acid catabolism. Importantly, however, blocking aerobic fatty acid utilization with etomoxir inhibited DAF-12-induced reproductive growth in *S*. *stercoralis* (Fig. 5B-D). Inhibiting this metabolic pathway in other parasitic species using this type of strategy has recently been suggested [41]. To date, there is a very limited armamentarium of anthelmintic drugs that is effective against *S*. *stercoralis*, which can cause disseminated strongyloidiasis and multi-organ failure in infected humans. Although further studies in relevant host species are needed, our results suggest targeting metabolic enzymes may lead to a therapeutic approach for treating diseases caused by *S*. *stercoralis* and possibly other parasites [42]. To that end, it is interesting that etomoxir and other drugs that were originally developed to regulate fatty acid metabolism [37] as a means for treating diabetes and metabolic disease might be repurposed for treating parasitism.

In summary, our studies reveal a novel facet of DAF-12 activity in both *C*. *elegans* and parasitic nematodes, namely the regulation of fatty acid catabolism and energy homeostasis. In this regard, DAF-12 is similar to the PPAR subfamily of nuclear receptors, which coordinately regulate fatty acid homeostasis and energy balance in vertebrates in response to nutrient availability. Our results provide a molecular explanation for how nematodes adjust energy homeostasis in response to changes in environmental conditions for reproduction. Moreover, they suggest a new strategy for developing new classes of anthelmintic drugs.

## Materials and Methods

### Reagents and nematode strains

Δ7-DA was synthesized as described [6]. *C*. *elegans* strains *din-1;daf-9(dh127;dh6)*, *din-1;daf-12(dh127;rh61rh411)* and *daf-9(dh6)* were from Dr. Adam Antebi (Max-Planck Institute for Aging); wild type (*N2* strain), *daf-2(e1368)* and *daf-7(e1372)* worms were from *C*. *elegans* Genome Center (University of Minnesota). *daf-7;daf-12(e1372;rh61h411)* mutant was made by crossing *daf-7* hermaphrodites with hemizygous *daf-12* males and was screened for dauer defective F2 progenies. The wild type (UPD) strain of *S*. *stercoralis*, used for developmental switching studies, and an iso-female line (PV001) of this parasite, used for qPCR studies, were maintained as described [43].

### Lipid metabolism

Vehicle or Δ7-DA was mixed with 5× concentrated OP50 bacteria culture and loaded on NGM-agar plates. L1 larvae prepared by egg synchronization were cultured on these plates at 25°C for 22.5 h and the resulting L3 larvae were collected and washed in M9 buffer for the indicated assays. For triglyceride (TG) content, worms were sonicated and the resulting lysates were centrifuged at 13000×g at 4°C. From supernatants, total glyceride (TGs plus free glycerol) and free glycerol were measured by Infinity TG Reagent (Thermo Sci) and Free Glycerol Reagent (Sigma), respectively. TG levels were calculated by subtracting free glycerol from total and were normalized to protein amounts in the lysates.

Fatty acid oxidation was measured as described by the production of H_2_O from fatty acid [44]. Briefly, the L3 larvae from different treatment groups were incubated with a mix of cold and [^3^H]-palmitic acid (Perkin Elmer) complexed with fatty acid-free BSA (Sigma), and incubated in a shaker at 25°C for 1 h. The reaction was terminated by adding 10% TCA followed by centrifugation at 13000×g for 5 min to obtain supernatant. Remaining [^3^H]-palmitic acid was deprotonated by adding 5N NaOH and PBS and removed by ion exchange column (Dowex 1×8 200–400 mesh Cl, strongly basic, Sigma). [^3^H]_2_O left in the supernatant was measured by scintillation counting.

For oxygen consumption, worms were mixed with antibiotic-killed OP50 bacteria, and then transferred to Oxygen Biosensor Plates (BD Bioscience) for oxygen measurement. Oxygen consumption was expressed as the increase of fluorescence units (ΔFU) and was normalized by protein amounts of the worms. For fatty acid uptake, worms were mixed with OP50 bacteria and 250 nM of fluorescent tracer (C_1_-BODIPY-C_12_ fatty acid, Invitrogen). Following 1 h incubation at 25°C, worms were washed, mounted, and photographed under fluorescence microscopy. Fluorescence density units (FU) of each worm were quantified by the software Image-J.

### Measurement of pharyngeal pumping rates

Pharyngeal pumping rates were measured as described [45]. Briefly, *din-1;daf-9* L3 larvae were transferred to a fresh NGM plate with OP50 bacteria lawn and were videotaped through a stereomicroscope. Pumping rates were measured by counting the grinder movements and presented as pumps per minute. For each treatment, 10 L3s were assayed.

### Reproductive growth assays

Reproductive growth of *C*. *elegans* was measured by L4-young adult transition or by egg laying assays. For L4-YA transition assay, synchronized L1 larvae from were cultured on NGM-agar plates pre-loaded with 5× concentrated HT115 bacteria culture. Worms were grown at 20°C and young adults were counted at indicated time points. Data were presented as the percentage of young adults in whole populations. For the egg-laying assay, synchronized *din-1;daf-9* L1 larvae were grown at 25°C on NGM-agar plates pre-loaded with 5× concentrated OP50 bacteria culture. At the indicated time points, 10~15 worms were transferred to fresh plates with bacterial lawns for 2.5 h and laid eggs were counted. Data were presented as numbers of the eggs laid by each worm per hour.

### Quantitative RT-PCR (qPCR) and microarray analysis

For *C*. *elegans* experiments, synchronized L1 larvae were treated with vehicle or Δ7-DA for 22.5 h at 25°C, or synchronized wild type L1 larvae were cultured to L4 stage and then harvested as fed worms or were deprived of food for an additional 12 h to obtain fasted worms. For *S*. *stercoralis* experiments, iL3s worms were treated with or without Δ7-DA in M9 buffer at 37°C and 5% CO_2_ in air for 24 h. Total RNA from worms was extracted with TRIzol Reagent (Life Technologies), and analyzed by qPCR. Relative mRNA levels were normalized to expression of reference genes *inf-1* or *ama-1 (C*. *elegans)* or 18S ribosomal RNA *(S*. *stercoralis)*. Data were presented as fold changes of relative mRNA levels in DA versus vehicle treated worms or in fasted versus fed worms.

Total RNA was also subjected to the *C*. *elegans* Genome Array (Affymetrix) for whole genome gene expression analysis. Briefly, gene expression values were log2 transformed and genes with >10-fold difference between replicates in either of the treatments were removed from our analysis. To identify the differentially expressed genes, we applied Significance Analysis of Microarrays (SAM) analysis using the R package samr [46]. Genes with median false discovery <5% and fold changes >2.0 were considered differentially expressed.

### Electrophoretic mobility shift assay

DAF-12 proteins were prepared with TNT Quick-Coupled Transcription/Translation System (Promega) and blocked with poly-[dI-dC] and non-specific single-stranded oligos. The DAF-12 proteins were then incubated with [^32^P]-end-labeled dsDNA probes (S3 Table) at room temperature for 30 min and binding to DAF-12 was analyzed by 5% PAGE followed by autography. For competitive binding experiments, 20- or 200-fold excesses of unlabeled DNA probes were also included in the binding reaction.

### Mammalian cell-based reporter assay

Co-transfection and luciferase reporter assays were performed as described in HEK 293 cells [15]. Eight hours post-transfection, cells were treated with vehicle or 1 μM Δ7-DA, and luciferase and β-galactosidase activities were then measured 16 h later. Relative luciferase units (RLU) were normalized to β-galactosidase activity. Reporter plasmids were constructed by inserting DAF-12REs and their 10-bp genomic flanking sequences into a TK-luc reporter plasmid.

### Nuclear run-on assay

Synchronized *din-1;daf-9* L1 larvae were grown in 5×concentrated OP50 in liquid suspension and shaken at 25°C for 15 h. Resulting L2 larvae were treated transiently with vehicle or 500 nM Δ7-DA and harvested in ice-cold M9 buffer. Cell nuclei were extracted and incubated with ATP, CTP, GTP and 5’-Bromo-UTP (BrUTP) at 30°C for labeling of nascent RNAs with BrUTP (BrUTP-RNAs). The BrUTP-RNAs were then enriched with anti-BrUTP agorase beads (Santa Cruz) and quantified by qPCR.

### Development switching assays

Development switching assays were performed as described [5,15]. Briefly, synchronized L1 larvae (*C*. *elegans*) or eggs (*S*. *stercoralis*) were grown on NGM-plates pre-loaded with etomoxir and phenotypes were observed after 60 h incubation at 25°C. Data from three independent experiments were pooled and significance was determined by Fisher’s exact test.

### *S*. *stercoralis* gene homolog identification

*S*. *stercoralis* homologs were identified as reported [43] by a TBLASTN (NCBI) search of *C*. *elegans* versus *S*. *stercoralis* (6 December 2011 draft; ftp://ftp.sanger.ac.uk/pub/pathogens/HGI/) databases, followed by annotation to RNA-seq data (ArrayExpress accession number E-MTAB-1164). Phylogenetic tree analyses were constructed to resolve gene homology. *S*. *stercoralis* genes with 1:1 homology to *C*. *elegans* genes were identified as homologous genes.

### Statistical analysis

Unless otherwise stated, data were expressed as mean ± SD or SEM and significance tests between vehicle- or DA-treated groups were performed by Student’s t-test. The statistic tests of overlap between two gene sets were based on hypergeometric distribution and calculated by the R function “phyper ()” (https://stat.ethz.ch/R-manual/R-patched/library/stats/html/Hypergeometric.html).

## Supporting Information

S1 Fig Comparison of the DAF-12 transcriptome with the dauer recovery and DAF-16 regulated transcriptomes. (A) Comparative expression of genes up-regulated by DA and by exit from dauer. (B, C) Comparative expression of genes regulated by DA activation of DAF-12 and by the activation of DAF-16. The *P*-values were obtained through statistical tests of overlapping based on hypergeometric distribution.
(TIF)

S2 Fig Feedback inhibition of DAF-12 endocrine signaling pathway. RNA expression from synchronized L3 larvae were collected for qPCR analysis. Samples were from the same experiment shown in Fig. 2. *, *P* < 0.05 by Student’s t-test; n.s., not significant; n = 4 ± S.D.
(TIF)

S3 Fig Etomoxir treatment inhibits fatty acid oxidation in *C*. *elegans*. Synchronized *N2* or *din-1;daf-9* L1 worms were liquid cultured in S-medium and treated with 40 μM etomoxir at 25° C for 24 h in presence of food. Fatty acid oxidation (A, B) or triglyceride content assay (C) were then measured as described in methods. *, *P* < 0.05 by Student’s t-test; n = 3 ± S.D. in (A, C) and n = 5 ± S.D. in (B).
(TIF)

S4 Fig The effect of etomoxir on reproductive growth. Synchronized L1 larvae from N2 and *din-1;daf-12*, *daf-9;daf-12* and *daf-7;daf-12* mutants were treated with vehicle (ethanol) or 200 nM DA with or without 40 μM etomoxir. Egg-laying assays then performed following 48 h and 72 h (A–C) or 70 and 94 h (D) of incubation at 25° C in presence of food. **, *P* < 0.01 by Student’s t-test; n = 3 ± S.D.; n.s., not significant.
(TIF)

S1 Table Affymetrix microarray analysis of DAF-12 regulated genes. Synchronized *din-1;daf-9* L1s were treated with vehicle (ethanol) or 200 nM DA for 22.5 h at 25°C in presence of food. RNA from the resulting worms were extracted and microarray analysis (Affymetrix platform) performed.
(XLSX)

S2 Table Changes in expression of metabolic genes regulated by DA and fasting. Synchronized *din-1;daf-9* L1s were treated with vehicle (ethanol) or 200 nM DA for 22.5 h at 25°C in presence of food. RNA from the resulting worms were extracted and qPCR was then performed. The samples are same as in Fig. 2.
(DOCX)

S3 Table Characterization of DAF-12REs on transcription regulatory regions of fat utilizing genes. The sequences and chromosomal locations of DAF-12 REs as well as their activities for DAF-12 binding and inducing luciferase reporter expression are listed.
(DOCX)

S4 Table Regulation of fat utilization genes by DAF-12 in *S*. *stercoralis*. Infectious L3s of *S*. *stercoralis* were treated with or without Δ7-DA in M9 buffer at 37°C and 5% CO_2_ in air for 24 h. Total RNA from worms was extracted and analyzed by qPCR. The samples are same as in Fig. 5A.
(DOCX)
