# Supplementary figures and images for: The Nuclear Receptor DAF-12 Regulates Nutrient Metabolism and Reproductive Growth in Nematodes
Source: PLoS Genet. 2015 Mar 16;11(3):e1005027. doi: 10.1371/journal.pgen.1005027 (PMC4361679; doi:10.1371/journal.pgen.1005027)

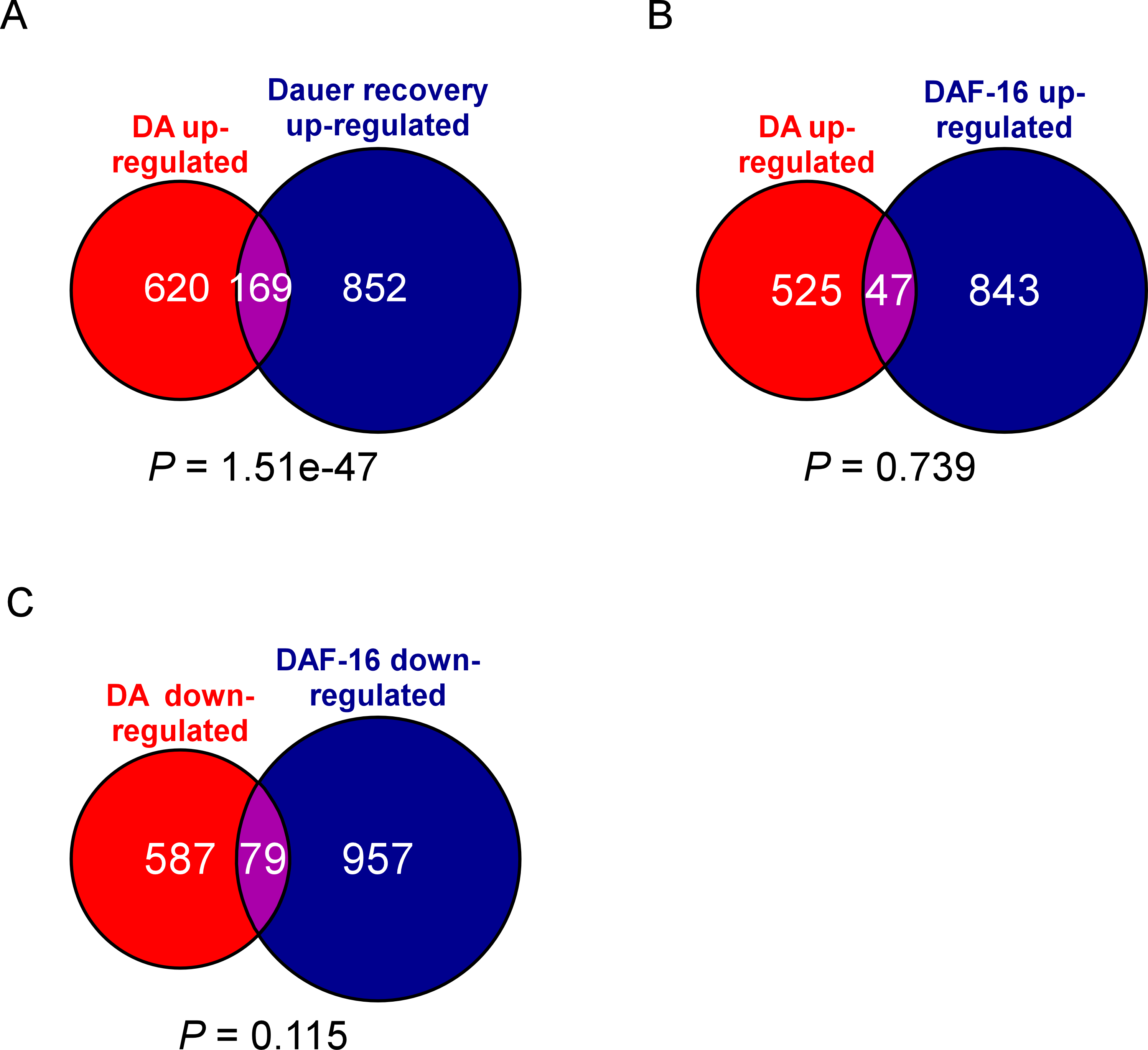

Supplement: S1 Fig — (A) Comparative expression of genes up-regulated by DA and by exit from dauer. (B, C) Comparative expression of genes regulated by DA activation of DAF-12 and by the activation of DAF-16. The P-values were obtained through statistical tests of overlapping based on hypergeometric distribution. (TIF) [file pgen.1005027.s001.tif]

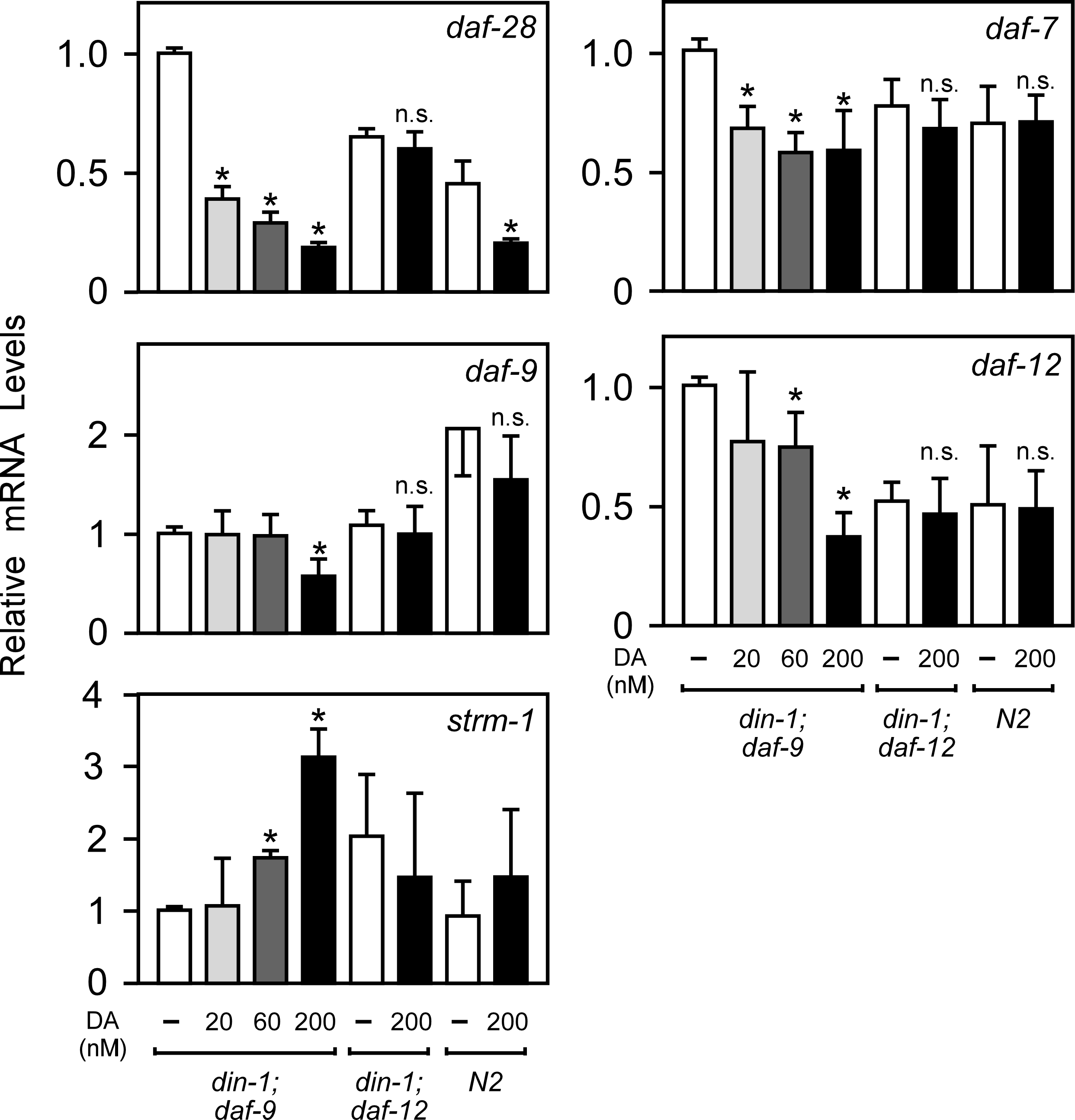

Supplement: S2 Fig — RNA expression from synchronized L3 larvae were collected for qPCR analysis. Samples were from the same experiment shown in Fig. 2. *, P < 0.05 by Student’s t-test; n.s., not significant; n = 4 ± S.D. (TIF) [file pgen.1005027.s002.tif]

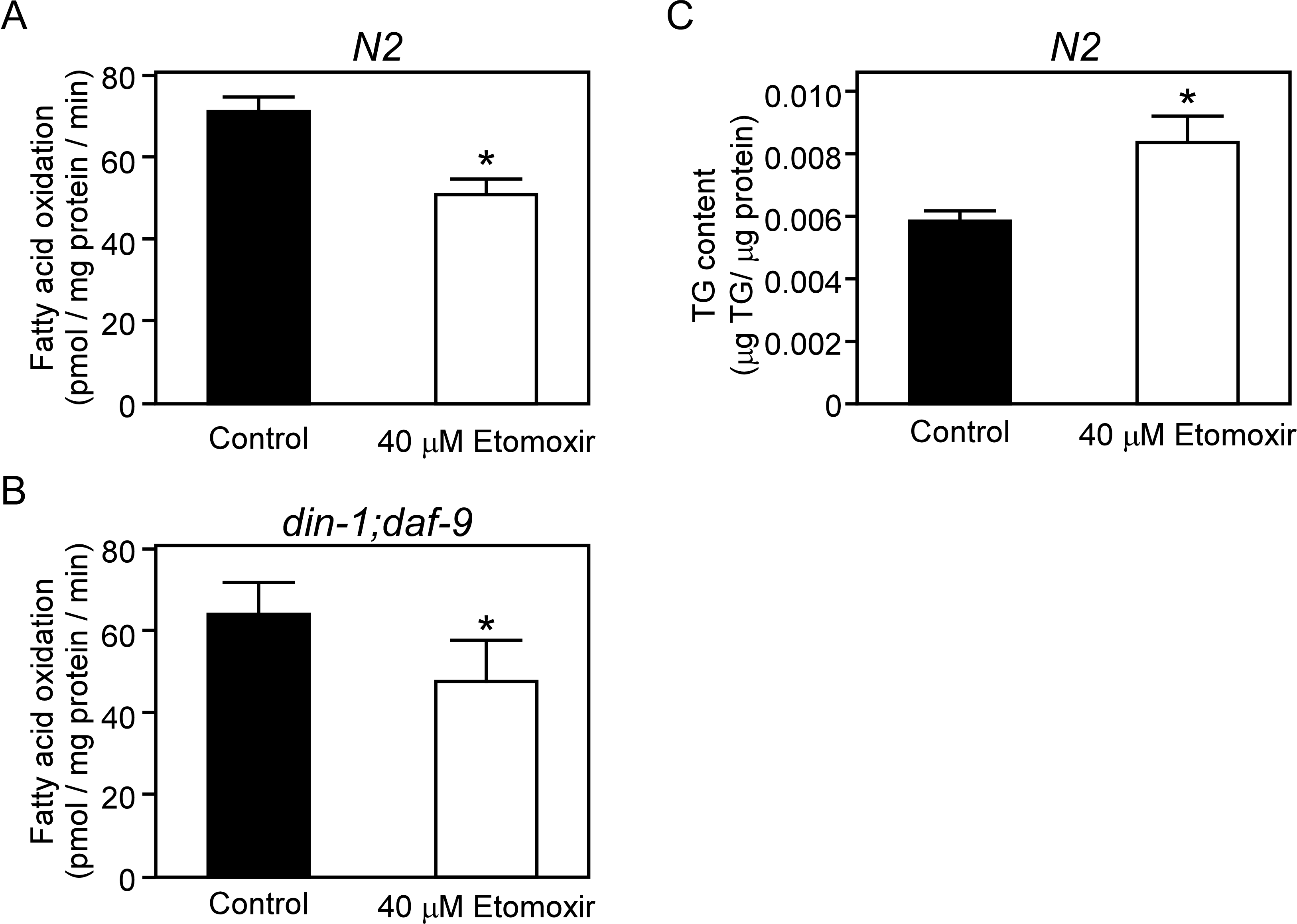

Supplement: S3 Fig — Synchronized N2 or din-1;daf-9 L1 worms were liquid cultured in S-medium and treated with 40 μM etomoxir at 25° C for 24 h in presence of food. Fatty acid oxidation (A, B) or triglyceride content assay (C) were then measured as described in methods. *, P < 0.05 by Student’s t-test; n = 3 ± S.D. in (A, C) and n = 5 ± S.D. in (B). (TIF) [file pgen.1005027.s003.tif]

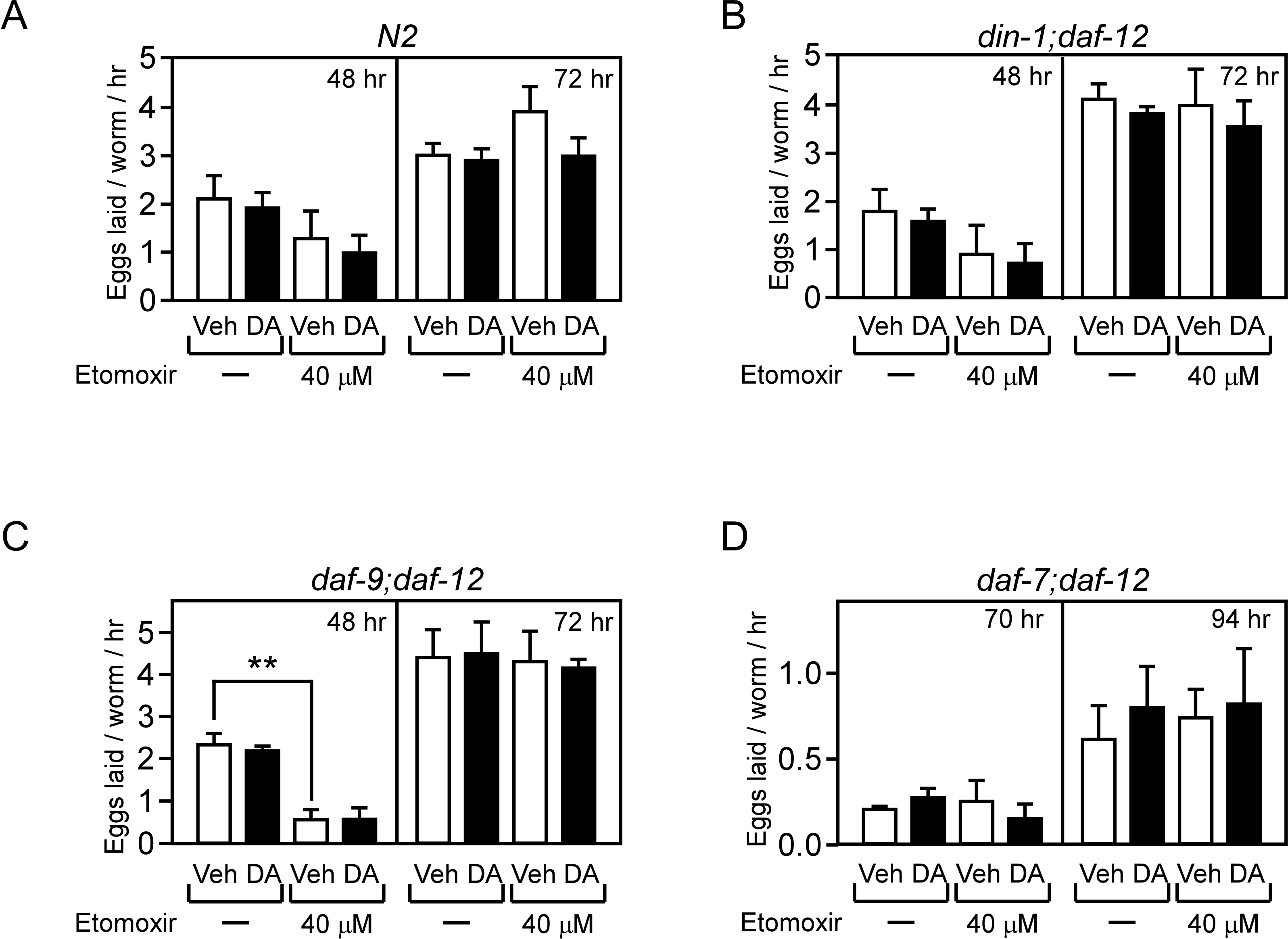

Supplement: S4 Fig — Synchronized L1 larvae from N2 and din-1;daf-12, daf-9;daf-12 and daf-7;daf-12 mutants were treated with vehicle (ethanol) or 200 nM DA with or without 40 μM etomoxir. Egg-laying assays then performed following 48 h and 72 h (A–C) or 70 and 94 h (D) of incubation at 25° C in presence of food. **, P < 0.01 by Student’s t-test; n = 3 ± S.D.; n.s., not significant. (TIF) [file pgen.1005027.s004.tif]
