# Supplementary material for: The Nuclear Receptor DAF-12 Regulates Nutrient Metabolism and Reproductive Growth in Nematodes
Source: PLoS Genet. 2015 Mar 16;11(3):e1005027. doi: 10.1371/journal.pgen.1005027 (PMC4361679; doi:10.1371/journal.pgen.1005027)
Supplement: S2 Table — Synchronized din-1;daf-9 L1s were treated with vehicle (ethanol) or 200 nM DA for 22.5 h at 25°C in presence of food. RNA from the resulting worms were extracted and qPCR was then performed. The samples are same as in Fig. 2. (DOCX) [file pgen.1005027.s006.docx]

**Table S2. Changes in expression of metabolic genes regulated by DA, dauer formation and fasting.**

1, "DA regulation" data were collected from qPCR measurements unless marked by *, indicating collection from microarray (Table S1). The values in each cell represent the folds of change in expression levels of the genes caused by 200 nM DA treatment.

2, "Fasting regulation" data were collected from qPCR experiments.

Red, up-regulated; Green, down-regulated; Grey, not changed.

| **Fatty acid metabolism** | |  |  |  |  |
| --- | --- | --- | --- | --- | --- |
| Gene name | Gene ID | Gene family | Gene function | DA regulation^1^ | Fasting regulation^2^ |
| *K08B12.1* | K08B12.1 | lipase | fatty acid mobilization | 13.75 | 82.03 |
| *hosl-1* | C46C11.1 | triglyceride lipase | fatty acid mobilization | 1 | 1 |
| *fil-1* | T01C3.4 | lipase | fatty acid mobilization | 0.37 | 11.46 |
| *lips-1* | F45E6.4 | lipase | fatty acid mobilization | 1 | 2.61 |
| *lips-3* | F10F2.3 | lipase | fatty acid mobilization | 1 | 18.89 |
| *lips-6* | ZK617.2 | lipase | fatty acid mobilization | 4.68 | 65.19 |
| *lips-7* | C09E8.2 | lipase | fatty acid mobilization | 1 | 22.36 |
| *lips-8* | F31F6.7 | lipase | fatty acid mobilization | 1 | 3.08 |
| *lips-9* | F58G1.5 | lipase | fatty acid mobilization | 3.47 | 16.21 |
| *lips-10* | F14E5.5 | lipase | fatty acid mobilization | 1 | 1 |
| *lips-11* | T13B5.5 | lipase | fatty acid mobilization | 0.28 | 7.98 |
| *lips-14* | H17B01.3 | lipase | fatty acid mobilization | 1 | 0.08 |
| *lips-15* | Y38E10A.7 | lipase | fatty acid mobilization | 1 | 2.46 |
| *lips-16* | Y38E10A.10 | lipase | fatty acid mobilization | 1 | 2.2 |
| *lips-17* | R07G3.2 | lipase | fatty acid mobilization | 0.09 | 0.52 |
| *lipl-1* | F54F3.3 | lipase | fatty acid mobilization | 1 | 1 |
| *lipl-2* | F46B6.8 | lipase | fatty acid mobilization | 1 | 5.03 |
| *lipl-3* | R11G11.14 | lipase | fatty acid mobilization | 1 | 31.28 |
| *lipl-4* | K04A8.5 | lipase | fatty acid mobilization | 1 | 15.45 |
| *lipl-5* | ZK6.7 | lipase | fatty acid mobilization | 1 | 2.27 |
| *lipl-6* | Y57E12B.3 | lipase | fatty acid mobilization | 3.85 | 10.17 |
| *lipl-7* | F01G10.7 | lipase | fatty acid mobilization | 1 | 1 |
| *K03H6.2* | K03H6.2 | lipase | fatty acid mobilization | 3.09 | 0.27 |
| *C40H1.7* | C40H1.7 | lipase | fatty acid mobilization | 2.85 | 1 |
| *F28H7.3* | F28H7.3 | lipase | fatty acid mobilization | 3.69 | 0.15 |
| *Y49E10.18* | Y49E10.18 | lipase | fatty acid mobilization | 3.7 | 1 |
| *F25A2.1* | F25A2.1 | lipase | fatty acid mobilization | 0.22 | 1 |
| *acs-1* | F46E10.1 | acyl-CoA synthetase | fatty acid esterification | 2.02 | 0.35 |
| *acs-2* | F28F8.2 | acyl-CoA synthetase | fatty acid esterification | 0.38 | 102.54 |
| *acs-3* | T08B1.6 | acyl-CoA synthetase | fatty acid esterification | 3.01 | 4.11 |
| *acs-4* | F37C12.7 | acyl-CoA synthetase | fatty acid esterification | 1 | 1 |
| *acs-5* | Y76A2B.3 | acyl-CoA synthetase | fatty acid esterification | 1 | 1 |
| *acs-11* | F41C3.3 | acyl-CoA synthetase | fatty acid esterification | 1 | 2.62 |
| *acs-20* | F28D1.9 | acyl-CoA synthetase | fatty acid esterification | 2.23 | 7.16 |
| *acbp-3* | F47B10.7 | acyl-CoA binding protein | fatty acid binding & transport | 2.52 | 2.19 |
| *lbp-1* | F40F4.3 | fatty acid binding protein | fatty acid binding & transport | 1 | 4.48 |
| *lbp-4* | ZK742.5 | fatty acid binding protein | fatty acid binding & transport | 1 | 1 |
| *lbp-5* | W02D3.7 | fatty acid binding protein | fatty acid binding & transport | 0.45 | 2 |
| *lbp-7* | T22G5.2 | fatty acid binding protein | fatty acid binding & transport | 0.48 | 1.93 |
| *lbp-8* | T22G5.6 | fatty acid binding protein | fatty acid binding & transport | 0.31 | 0.07 |
| *F08A8.2* | F08A8.2 | acyl-CoA oxidase, peroxisomal | peroxisomal fatty acid β-oxidation | 6.83 | 0.25 |
| *F08A8.1* | F08A8.1 | acyl-CoA oxidase, peroxisomal | peroxisomal fatty acid β-oxidation | 1 | 1 |
| *F08A8.4* | F08A8.4 | acyl-CoA oxidase, peroxisomal | peroxisomal fatty acid β-oxidation | 1 | 0.06 |
| *F59F4.1* | F59F4.1 | acyl-CoA oxidase, peroxisomal | peroxisomal fatty acid β-oxidation | 1 | 2.29 |
| *C48B4.1* | C48B4.1 | acyl-CoA oxidase, peroxisomal | peroxisomal fatty acid β-oxidation | 0.46 | 1 |
| *ech-8* | F01G10.2 | enoyl-CoA hydratase, peroxisomal | peroxisomal fatty acid β-oxidation | 1 | 3.61 |
| *ech-9* | F01G10.3 | enoyl-CoA hydratase, peroxisomal | peroxisomal fatty acid β-oxidation | 1 | 6.52 |
| *T05E7.1* | T05E7.1 | long chain fatty-CoA thiolase, peroxisomal | peroxisomal fatty acid β-oxidation | 2.16 | 0.58 |
| *cpt-1* | Y46G5A.17 | CPT-I | mitochondrial fatty acid β-oxidation | 1 | 1 |
| *cpt-2* | R07H5.2 | CPT-I | mitochondrial fatty acid β-oxidation | 1 | 1 |
| *cpt-3* | Y48G9A.10 | CPT-I | mitochondrial fatty acid β-oxidation | 0.43 | 109.97 |
| *cpt-4* | K11D12.4 | CPT-I | mitochondrial fatty acid β-oxidation | 1 | 5.57 |
| *cpt-5* | F09F3.9 | CPT-I | mitochondrial fatty acid β-oxidation | 2.49 | 0.3 |
| *cpt-6* | W01A11.5 | CPT-I | mitochondrial fatty acid β-oxidation | 4.39 | 1 |
| *acdh-2* | C17C3.12 | acyl-CoA dehydrogenase | mitochondrial fatty acid β-oxidation | 0.26 | 0.02 |
| *icl-1* | C05E4.9 | isocitrate lyase/malate synthase | glyoxylate cycle | 0.56 | 20.36 |
| *F48E8.3* | F48E8.3 | Fumarate Reductase | malate dismutation | 1 | 1.98 |
| *NHR-49* | K10C3.6 | HNF-4α, nuclear receptor | lipolysis | 1 | 1.65 |
| *sbp-1* | Y47D3B.7 | SREBP-1c | lipogenesis | 0.48 | 1 |
| *FASN-1* | F32H2.5 | fatty acid synthase | fatty acid biosynthesis | 2.92 | 1 |
| *F32H2.6* | F32H2.6 | fatty acid synthase | fatty acid biosynthesis | 2.46 | 6.01 |
| *pod-2* | W09B6.1 | acyl-CoA carboxylase | fatty acid biosynthesis | 1 | 1 |
| *fat-2* | W02A2.1 | fatty acid desaturase | fatty acid desaturation | 1 | 1 |
| *fat-3* | W08D2.4 | fatty acid desaturase | fatty acid desaturation | 1 | 3.27 |
| *fat-5* | W06D12.3 | fatty acid desaturase | fatty acid desaturation | 1 | 1 |
| *fat-6* | VZK822L.1 | fatty acid desaturase | fatty acid desaturation | 1 | 3.31 |
| *fat-7* | F10D2.9 | fatty acid desaturase | fatty acid desaturation | 12.85 | 0.03 |
| *Y54E5A.1* | Y54E5A.1 | fatty acid desaturase | fatty acid desaturation | 1 | 1.91 |
| *elo-4* | C40H1.4 | fatty acid elongase | fatty acid elongation | 4.93 | 8.79 |
|  |  |  |  |  |  |
| **Glucose metabolism** | |  |  |  |  |
| Gene name | Gene ID | Gene Family | Gene Function | DA regulation^1^ | Fasting regulation^2^ |
| *R11A5.4* | R11A5.4 | phosphoenolpyruvate carboxykinase | gluconeogenesis | NC* | 2.29 |
| *W05G11.6* | W05G11.6 | phosphoenolpyruvate carboxykinase | gluconeogenesis | NC* | 2.31 |
|  |  |  |  |  |  |
| **DAF-12 signaling pathway** | |  |  |  |  |
| Gene name | Gene ID | Gene Family | Gene Function | DA regulation^1^ | Fasting regulation^2^ |
| *daf-28* | Y116F11B.1 | insulin/IGF-I | insulin signaling pathway | 0.19 |  |
| *daf-7* | B0412.2 | TGF-beta | TGF-beta signaling pathway | 0.59 |  |
| *daf-12* | F11A1.3 | Nuclear receptor | dauer regulation | 0.37 |  |
| *daf-9* | T13C5.1 | CYP450 | dauer regulation | 0.58 |  |
| *strm-1* | H14E04.1 | SAM-dependent methyltransferases | dauer regulation | 3.14 |  |
