# Supplementary material for: The Nuclear Receptor DAF-12 Regulates Nutrient Metabolism and Reproductive Growth in Nematodes
Source: PLoS Genet. 2015 Mar 16;11(3):e1005027. doi: 10.1371/journal.pgen.1005027 (PMC4361679; doi:10.1371/journal.pgen.1005027)
Supplement: S3 Table — The sequences and chromosomal locations of DAF-12 REs as well as their activities for DAF-12 binding and inducing luciferase reporter expression are listed. (DOCX) [file pgen.1005027.s007.docx]

**Table S3. Characterization of DAF-12REs on transcription regulatory regions of fat utilizing genes**

a, Putative DAF-12REs are highlighted in uppercase letters. b, the position is relative to translation start sites (the transcription start sites have not been well-characterized for these genes). c, DAF-12 binding to the response element were determined by EMSA. d, reporter activity was determined by cell-based reporter assay as described in methods (n.d. not done).

| Putative REs | Sequences^a^ | Position^b^ | DAF-12 Binding^c^ | Reporter  activity^d^ |
| --- | --- | --- | --- | --- |
| *acs-1 gene* |  |  |  |  |
| *acs-1a* | ttcatcataaAGTGCAtctctatttt | -2230 | YES | YES |
| *acs-1b* | tggtttagtcAGTTCAgaaatgaacc | -1492 | NO | n.d |
| *acs-1c* | gttggaaaaaAGTACAttaaataatt | -903 | YES | NO |
| *acs-1d* | atgtataattAGGACAtctgttctac | 270 | NO | n.d |
| *acs-1e* | aagttttttaAGTGCAtttttattgt | 1499 | YES | NO |
| *acs-1f* | tcccgatcttAGTTCAgatgactctt | 1927 | NO | n.d |
| *acs-1g* | aaaatcacaaAGTGCAaaatgaaaaa | 2511 | YES | YES |
| *acs-1h* | ctatagtctcAGTACAttctagccct | 2659 | NO | n.d |
| *acs-1i* | ctttacaaacAGTACAaacttttttt | 2911 | NO | n.d |
| *acs-1DR4* | aagataacgcAGTGCAaatgAGTGGAattgaaaaaa | 3048 | NO | n.d |
| *cpt-6 gene* |  |  |  |  |
| *cpt-6a* | attgtttacaAGTGCAgccccagtat | -2052 | YES | NO |
| *cpt-6IR9* | ataattttccAGTGCGtttttaagcTGAACTttcatttgaa | -1516 | NO | n.d |
| *cpt-6ER10* | aggtaattttAGCACTggttttaaacAGTACTcccaattctt | -1193 | NO | n.d |
| *cpt-6b* | tatccagtat tgtact tatgtttgta | -2516 | NO | n.d |
| *cpt-6c* | aaaaacagtt tgaact ggtaacaaat | -2442 | NO | n.d |
| *cpt-6d* | caatacaaac tgaact aattaaagat | -2398 | NO | n.d |
| *cpt-6e* | ttaaagtttg tgcact ttccattatc | -1965 | YES | n.d |
| *cpt-6f* | cggcggttat tgtact tttataattt | -1530 | YES | n.d |
| *cpt-6g* | gcggcatgtt tgaact tttttttata | -1216 | NO | n.d |
| *K08B12.1 gene* |  |  |  |  |
| *K08B12.1a* | cgatattgtaAGTACAaacctcttcc | -2299 | YES | NO |
| *K08B12.1b* | gtgcacaatgAGTACAagagacagtc | -1695 | NO | n.d |
| *K08B12.1c* | acgcacaagaAGTACAaagttgatga | -1592 | YES | YES |
| *K08B12.1d* | gatgagggggAGGGCAccagatatga | -1563 | NO | n.d |
| *K08B12.1e* | tcgacgctacAGTACTcatgtaaagt | -1105 | NO | n.d |
| *K08B12.1f* | tccgtccatcAGTGCTtccagatgcc | -431 | NO | n.d |
| *T05E7.1 gene* |  |  |  |  |
| *T05E7.1a* | agaatgggttAGTACAatataatcta | -1994 | NO | n.d |
| *T05E7.1b* | catttgaagaAGTGCGatctccgatt | -1892 | NO | n.d |
| *T05E7.1c* | aatgttaggtAGTTCAttacagcgtc | -921 | NO | n.d |
| *T05E7.1ER2* | ggttgcacgtTGTCCTagAGTTCAaacaacttac | -1962 | NO | n.d |
| *acs-3 gene* |  |  |  |  |
| *acs-3a* | caattaaaatAGTGCAtacaatagct | -4522 | YES | NO |
| *acs-3b* | ctttttataaAGTACAtttcctcatt | -3496 | YES | YES |
| *acs-3c* | tgaaggtaaaAGTGCAttatggttag | -3079 | YES | NO |
| *acs-3d* | gtactgtaaaAGTTCAacaaagcgtt | -2803 | YES | NO |
