# Supplementary material for: The Nuclear Receptor DAF-12 Regulates Nutrient Metabolism and Reproductive Growth in Nematodes
Source: PLoS Genet. 2015 Mar 16;11(3):e1005027. doi: 10.1371/journal.pgen.1005027 (PMC4361679; doi:10.1371/journal.pgen.1005027)
Supplement: S4 Table — Infectious L3s of S. stercoralis were treated with or without Δ7-DA in M9 buffer at 37°C and 5% CO2 in air for 24 h. Total RNA from worms was extracted and analyzed by qPCR. The samples are same as in Fig. 5A. (DOCX) [file pgen.1005027.s008.docx]

**Table S4. Regulation of fat utilization genes by DAF-12 in *S. stercoralis.*** The identification of *S. stercoralis* homologs of metabolic genes and the qPCR experiment was described in methods. a, Data were collected from qPCR measurements and represent fold-induction by DA relative to untreated worms. Red, up-regulated by DA; Green, down-regulated by DA; Grey, not changed (NC).

| Gene name | Genomic location | Homolog in *C. elegans* | Gene family | Gene function | DA induction in *S. stercoralis*^a^ |
| --- | --- | --- | --- | --- | --- |
| *Ss_K08B12.1* | contig-79597:97096-98281 | *K08B12.1* | lipase | fatty acid mobilization | NC |
| *Ss_F28H7.3* | contig-177448:78204-79257 | *F28H7.3* | lipase | fatty acid mobilization | 4.47 |
| *Ss_lipl-6* | contig-12406:2917-4576 | *lipl-6* | lipase, LPL family | fatty acid mobilization | 0.53 |
| *Ss_acs-1* | contig-135023:25616-27677 | *acs-1* | acyl-CoA synthetase | fatty acid esterification | 20.36 |
| *Ss_acs-2* | contig-160786:54950-56761 | *acs-2* | acyl-CoA synthetase | fatty acid esterification | 0.50 |
| *Ss_acs-5* | contig-214868:42643-44977 | *acs-5* | acyl-CoA synthetase | fatty acid esterification | NC |
| *Ss_acs-20* | contig-101420:712-3450 | *acs-20* | acyl-CoA synthetase | fatty acid esterification | 0.33 |
| *Ss_acs-22* | contig-101420:712-3450 | *acs-22* | acyl-CoA synthetase | fatty acid esterification | NC |
| *Ss_acbp-3* | contig-62425:45343-45969 | *acbp-3* | acyl-CoA binding protein | fatty acid binding & transport | 6.79 |
| *Ss_acox-1* | contig-135023:85061-87095 | *related to F08A8.2* | acyl-CoA oxidase, peroxisomal | peroxisomal fatty acid β-oxidation | NC |
| *Ss_acox-2* | contig-5202:8787-10800 | *related to F08A8.2* | acyl-CoA oxidase, peroxisomal | peroxisomal fatty acid β-oxidation | 0.53 |
| *Ss_acox-3* | contig-48040:38251-40371 | *related to F08A8.2* | acyl-CoA oxidase, peroxisomal | peroxisomal fatty acid β-oxidation | 0.37 |
| *Ss_ech-8* | contig-20884:28105-30092 | *ech-8* | enoyl-CoA hydratase, perioximal | peroxisomal fatty acid β-oxidation | 0.12 |
| *Ss_cpt-1* | contig-103122:32076-34493 | *cpt-1* | CPT-I | mitochondrial fatty acid β-oxidation | NC |
| *Ss_cpt-2* | contig-12351:14385-16406 | *cpt-2* | CPT-I | mitochondrial fatty acid β-oxidation | 0.45 |
| *Ss_cpt-3* | contig-62379:2516-4794 | *no clear homolog* | CPT-I | mitochondrial fatty acid β-oxidation | NC |
| *Ss_W03F9.4* | contig-50770:40277-42545 | *W03F9.4* | CPT-I | mitochondrial fatty acid β-oxidation | 2.49 |
| *Ss_gei-7* | contig-135023:68455-71527 | *gei-7* | isocitrate lyase/malate synthase | glyoxylate cycle | 0.34 |
| *Ss_F48E8.3* | contig-44516:46774-48304 | *F48E8.3* | fumarate reductase | malate dismutation | NC |
